# A bibliometric and emerging trend analysis on stress granules from 2011 to 2020: A systematic review and bibliometrics analysis

**DOI:** 10.1097/MD.0000000000029200

**Published:** 2022-07-22

**Authors:** Haiyang Yu, Qinhao Chen, Yueyin Pan

**Affiliations:** a Provincial Hospital Affiliated to Anhui Medical University, Hefei, Anhui Province, China; b Wannan Medical College, Wuhu, Anhui Province, China; c The First Affiliated Hospital of University of Science and Technology of China, Hefei, Anhui Province, China.

**Keywords:** bibliometrics, imaging, phase separation, RNP granule, stress granules

## Abstract

Stress granules (SGs) are the dense granules formed in the cytoplasm of eukaryotic cells in response to stress stimuli, such as endoplasmic reticulum stress, heat shock, hypoxia, and arsenate exposure. Although SGs have been attracting a lot of research attention, there is still a lack of systematic analysis of SGs in the literature.

**Methods:**

By analyzing the literature published in the Web of Science database using the R software, we extracted all the information related to SGs from the literature and cited references. The following information was included: publications per year, overall citations, top 10 countries, top 10 authors, co-author collaborations, top 10 institutions, critical areas, and top 10 cited research articles.

**Results:**

A total of 4052 articles related to SGs were selected and screened. These documents have been cited a total of 110,553 times, with an H-index of 126 and an average of 27.28 citations per article. The authors of the literature included in this study were from 89 different countries/regions. The United States and China had the highest number of publications and ranking institutions.

**Conclusions:**

This article presents essential insights on the characteristics and influence of SGs, demonstrating their indispensable role in immune regulation and other fields.

## 1. Introduction

The term “stress granules” (SGs) was first used in 1988 to describe a dense body formed in the cytoplasm of chicken embryonic fibroblasts under stimulation or stress.^[1]^ SGs are cytoplasmic aggregates containing protein and mRNA in the cytoplasm. They contain various translation initiation factors, RNA-binding proteins, and non-RNA-binding proteins and are formed by stagnant mRNA during initiation of translation. Cells try to avoid the adverse consequences induced by excessive adverse environmental conditions, such as heat shock, chemical exposure, oxidative stress, and aging, by producing SGs to maintain internal balance.^[2–6]^ Moreover, when the unfavorable conditions or stimuli disappear, the SGs existing in the cells will decompose and disappear accordingly. To enhance their ability to survive, cells adaptively produce cytoprotective proteins by regulating the formation and decomposition of SGs. In SGs, to meet the requirements for cell survival, necessary transcription sequences are prioritized and non-essential transcription sequences are suppressed temporarily.^[7,8]^

Arimoto et al have shown that different types of stress can lead to different types of responses, which can generally be divided into Type I and Type II stress. Type I stress refers to the preferential formation of SGs under stress conditions, such as arsenic exposure, heat shock, and hypoxia. Type II stress refers to the induction of SG formation by genotoxic drugs, such as etoposide and methyl methane sulfonate, through the activation of the stress-responsive mitogen-activated protein kinase cascade, and X-rays can also induce this type of stress response.^[9]^ SGs can inhibit mRNA degradation and temporarily store a part of the energy to provide the body with for mRNA re-synthesis.^[10]^ The functional response structure of SGs has also been reported. Multiple untranslated mRNAs together form an initiation complex. The mRNAs act like human bones to carry RNA-binding proteins. These proteins act through various protein–protein interactions.^[11–13]^ The internal and external environment of cells is complex and dynamic. As a coping method against stress, cells form SGs,^[14]^ and the decomposition and disappearance of SGs usually occur after the disappearance of stress and at the end of the translation process, after which the mRNA enters the translation multimer.^[15,16]^

As a membrane-less organelle, SGs are not only significant in the field of basic science, but they are also associated with the occurrence and progression of several kinds of diseases because of their functional properties. Previous studies have demonstrated the involvement of SGs in cancer, neurodegenerative diseases, and autoimmune diseases.^[17–19]^ There are many molecules involved in the signal transduction pathways related to the tumorigenesis process, some of which are involved in the formation of SGs.^[10,20]^ To a certain extent, the tumor microenvironment has an influence on the function of SGs, thereby promoting tumor growth and survival under a stressful environment.^[21–23]^ Gao X and other researchers showed that some chemotherapeutic drugs can induce the formation of SGs.^[10]^ Therefore, SGs may be a critical factor in cancer treatment in the future.^[24,25]^ The formation of SGs is inhibited when there is an interference in the formation and polymerization of microtubules, and with the inhibition of the SG synthesis pathways, SGs decrease or even disappear.^[10,21,26]^ The regulation of SGs can prevent the occurrence and deterioration of tumors, which shows that SGs have a wide range of potential application in the field of tumor treatment. The tumor microenvironment regulates many aspects of SGs, such as synthesis and degradation, through a complex mechanism of action, ultimately affecting tumor metastasis, drug resistance, and secondary mutations.^[10]^

According to Wei Q,^[27]^ the term bibliometrics is defined as “the application of mathematical and statistical methods to books and other communication media.” This definition lays the foundation for the writing of bibliometrics research and multi-disciplinary and multi-field research. Bibliometrics can be applied in all fields of study, and there are 2 common methods used in bibliometrics to track or predict development trends and hotspots in a research field, which are qualitative and quantitative methods. An increasing number of scholars are focusing on the research of pyrolysis, and it has also made significant leaps in the field of SGs. However, there is currently no report of the bibliometric summary of the literature on SGs. Cite Space and VOSviewer, 2 commonly used tools in bibliometrics, were used for statistical and visualization analysis of SGs research from 2005 to 2020 in this study. It is worth mentioning that this research provides some valuable directions and suggestions for future research work on SGs, by exploring hotspots and frontiers in the field.

## 2. Methods

### 2.1. Data sources and search strategy

Relevant publications from 2011 to 2020 were selected from the Web of Science database. To avoid output retrieval errors and ambiguities, data collection in this study was completed on November 11, 2021. The search subject was “Stress granules”; the language was limited to English, and the article type was limited to articles to avoid errors in the results caused by the inclusion of repeated studies. A total of 4052 articles meeting these retrieval conditions were selected for further analysis. The downloaded and retrieved materials included the record content of “full records and cited references”, and the file format of the materials was “plain text”. As only the file format named “download *.txt” can be recognized by Cite Space and VOSviewer,^[28]^ we renamed the files accordingly for further analysis. To avoid ambiguity caused by the database update on the research conclusions, the search was conducted within 1 day. The data used in this study were obtained from the WOS database, and hence, ethical approval does not apply to this research.

### 2.2. Data analysis and visualization

Data were processed using VOSviewer and Cite Space. The content of the publication included the year of publication, author, and country/region, which were standardized and unified for analysis using the bibliometric package in R 4.1.0. The calculation method of the journal impact factor for individual publications relied on the 2020 Journal Citation Report (JCR) (Clarivate Analytics, Philadelphia, USA). The relationships and network connections between things were determined by VOSviewer (version 1.6.16). We used CiteSpace version 5.8 to analyze and visualize the citation bursts, co-cited references, and high-frequency keyword trends. The operating parameters of Cite Space were set as follows: the number of years per slice (1), time span (2011–2020), and pruning (minimum spanning tree and pruning slice network), and other default settings were used.

## 3. Results

### 3.1. General statistics

**Figure 1.**
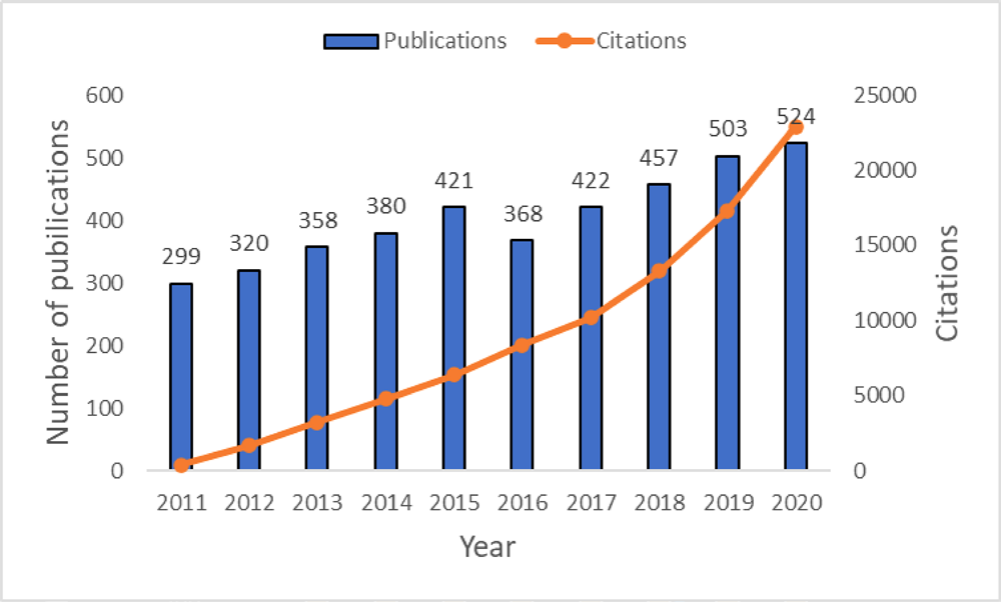
The number of publications in each time span on stress granules research from 2011 to 2020.

Based on the data collection strategy, 4052 papers, published between 2011 and 2020, were collected. SG-related references showed a high growth trend, from 299 articles (7.38%) in 2011 to 524 articles (12.93%) in 2020 (Fig. 1). All the included studies have been cited 110,553 times in total, with the average number of citations being 27.28 for each literature and an H-index of 126.

### 3.2. Active countries and institutions

**Figure 2.**
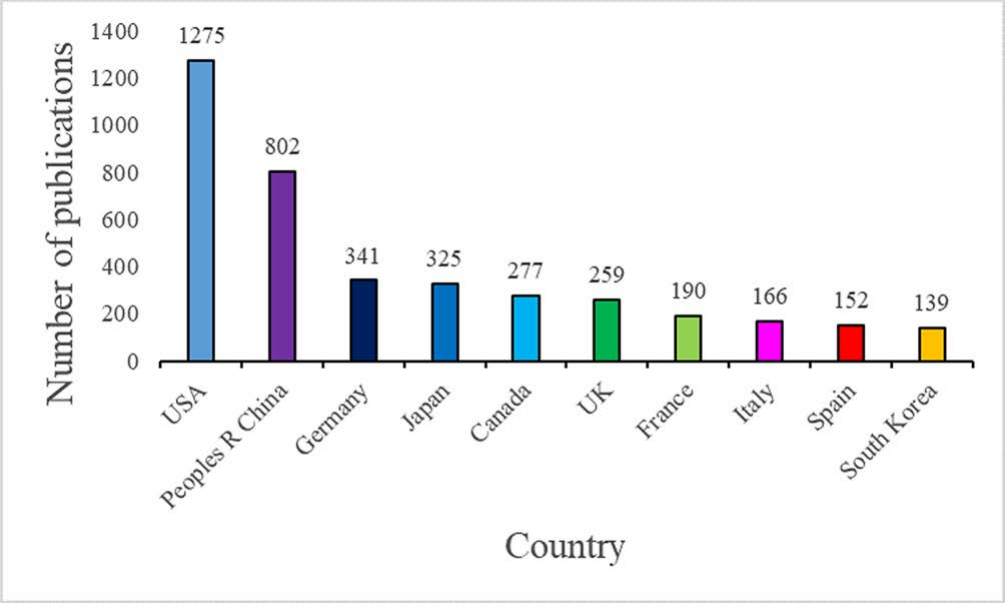
Top 10 countries with largest number of publications on stress granules research.

**Figure 3.**
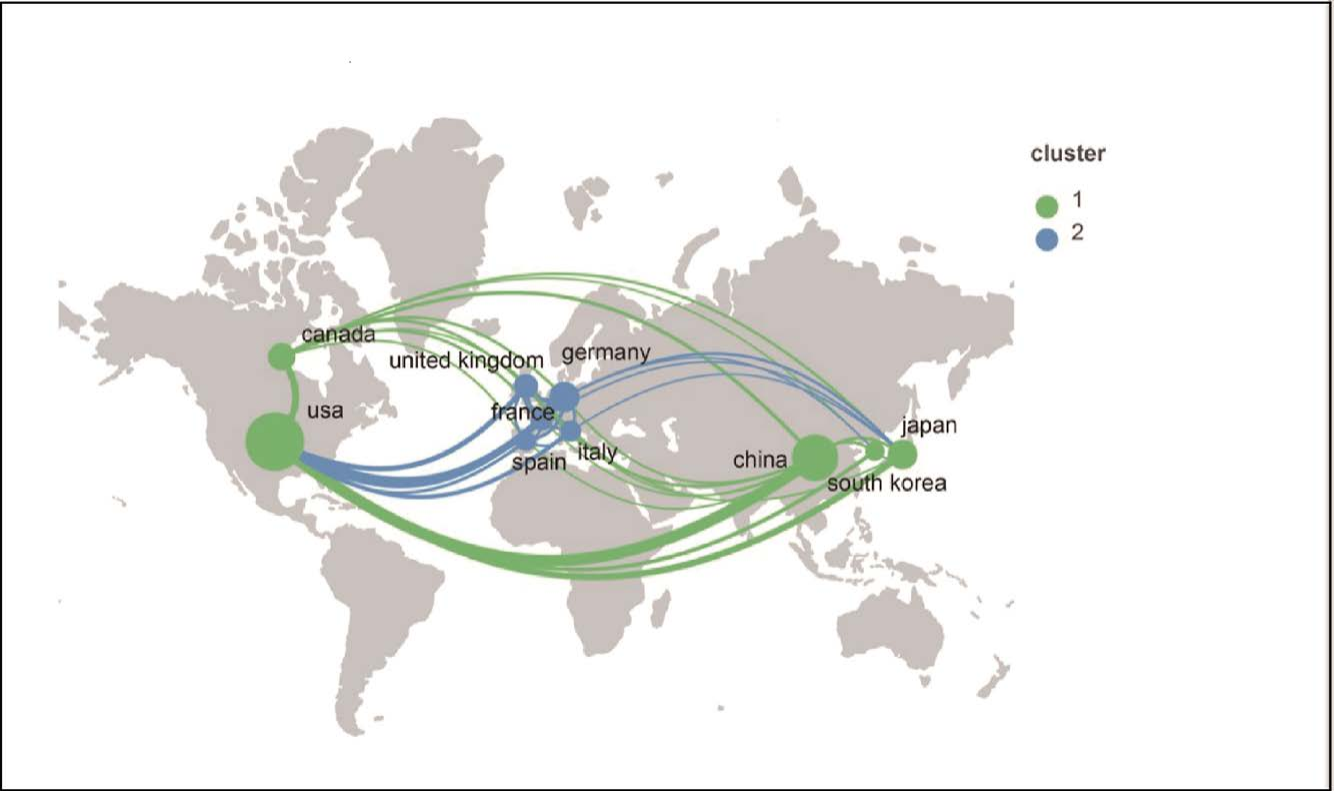
Major country/region partnerships for stress granules publications.

From 2011 to 2020 (Fig. 2), the United States had the highest contribution, with 1275 (31.47%) published literature, followed by China (802, 19.79%), Germany (341, 8.42%), Japan (325, 8.02%), and Canada (277, 6.84%). Given that they contribute more than 50% of the total number of papers published, both China and the United States can be regarded as the two dominant countries in the field of SG research. Co-country analysis showed a map of cooperative relations between various countries. The thickness of the country-to-country connection on the map indicated the closeness of cooperation (Fig. 3). A strong cooperation was observed between China and the United States. It is worth mentioning that the United States had strong collaborations with Germany and other countries, which illustrated that the United States had great attention to mutual cooperation.

Total global citation score means the total frequency of citation of an article in the current literature list in the Web of Science database, while total local citation score means the total frequency of citation of an article in the current literature list in other articles in the current literature list. CHINESE ACAD SCI published the highest number of literatures on SGs from 2011 to 2020 (69 publications), followed by McGill Univ, Univ Toronto, Univ Colorado, and Harvard Univ. Half of the ten institutions are in the USA, which indicates that this country is the pioneer in the field of SG research (Table 1).

**Table 1:** The top 10 institutions with stress granules publications.

No.	Institution	TLCS*	TGCS*	Records	Country
1	Chinese Acad Sci	51	1676	69	China
2	McGill Univ	303	2157	62	Canada
3	Univ Toronto	346	2833	48	Canada
4	Univ Colorado	875	4181	46	USA
5	Harvard Univ	481	3879	43	USA
6	Zhejiang Univ	22	1045	43	CHINA
7	Russian Acad Sci	86	835	40	Russian
8	Univ Penn	288	3267	40	USA
9	Brigham & Women's Hosp	425	2941	39	USA
10	Stanford Univ	389	2889	39	USA

* TGCS = total global citation score, TLCS = total local citation score.

### 3.3. Author analysis

**Figure 4.**
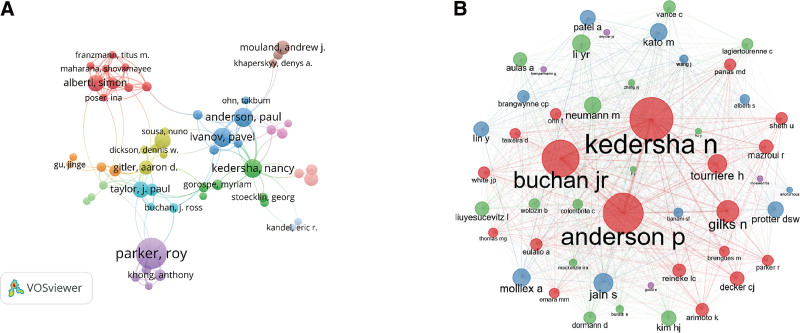
the network of authors in the stress granules field. (A) Cooperation network of authors (B)Co-citing authors cooperation network relationship.

Author analysis was performed for each author who published an article on the topic “SGs” in the past 10 years (Table 2). The results showed that Parker R, who published 40 articles in the field of SGs, was the most productive author. In addition, author Anderson P had a considerable contribution, given that he has published 23 articles on SGs. More detailed information about the number of papers published by the authors can be obtained from Table 2. In addition to Parker R and Anderson P, the top 5 authors included Li Y, Wang Y, and Li J. Moreover, Parker R ranked first in the total global citation score rankings (Table 2), followed by Taylor JP, Kim HJ, Alberti S, and Hyman AA. The VOSviewer was used to draw the cooperation network between authors, and Aiberti Simon (44 total link strength) showed the strongest cooperation with others among the authors included in our analysis, followed by Ivanov Pavel (40 total link strength) (Fig. 4).

**Table 2:** The top 10 authors in the stress granules field.

No.	Author	Recs	Percentage	Author	TGCS	Percentage
1	Parker R	40	0.99%	Parker R	4695	4.25%
2	Li Y	32	0.79%	Taylor JP	4404	3.98%
3	Wang Y	30	0.74%	Kim HJ	3714	3.36%
4	Li J	25	0.62%	Alberti S	3206	2.90%
5	Anderson P	23	0.57%	Hyman AA	2753	2.49%
6	Kedersha N	22	0.54%	Gitler AD	2638	2.39%
7	Ivanov P	21	0.52%	Hen R	2483	2.25%
8	Taylor JP	21	0.52%	Pappu RV	2471	2.24%
9	Wang J	20	0.49%	Molliex A	2231	2.02%
10	Wang X	20	0.49%	Maharana S	2215	2.00%

### 3.4. Journal analysis

In total, 1210 journals published SG-related publications. The top 10 journals published a total of 675 articles related to this field, accounting for 16.73% of all statistical data. The journal PLOS ONE (impact factor = 3.24, 2020) published the most articles related to SGs, with a total of 168 articles accounting for 4.15% of the total statistics (Table 3). The Journal of Virology published 88 papers, followed by Scientific Reports, which published 79 articles and Journal of Biological Chemistry, which published 78 papers. Six of the top 10 journals are in the United States, and four are in the United Kingdom. This is in line with the previous country analysis; that is, the United States still occupies a dominant position in the field of SGs.

**Table 3:** The top 10 journals in the stress granules field.

Journal	Recs	TGCS	Country	IF (2020)
PLOS ONE	168	4197	USA	3.24
JOURNAL OF VIROLOGY	88	2834	USA	5.10
SCIENTIFIC REPORTS	79	1270	UK	4.38
JOURNAL OF BIOLOGICAL CHEMISTRY	78	2403	USA	5.16
PNAS	50	2852	USA	9.58
JOURNAL OF CELL SCIENCE	48	1418	UK	5.29
NUCLEIC ACIDS RESEARCH	48	1577	UK	16.97
NATURE COMMUNICATIONS	44	1718	UK	14.92
CELL REPORTS	36	1511	USA	9.42
MOLECULAR CELL	36	4736	UK	27.40

### 3.5. Research area analysis

**Figure 5.**
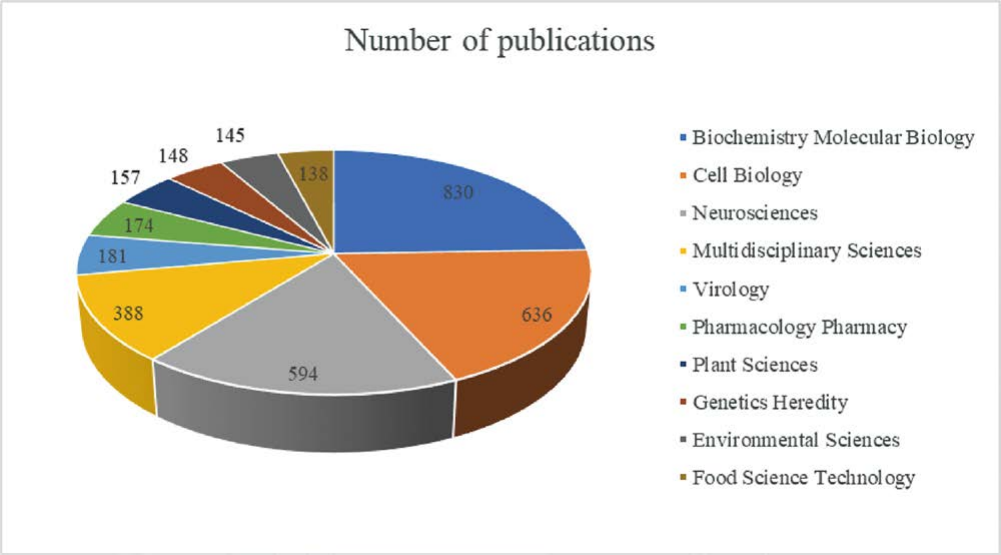
The top 10 research areas on stress granules research.

In more than 100 professional research fields, extensive investigations have been conducted on SGs. Further analysis revealed the top 10 research areas with the highest frequency of publications related to SGs research from 2011 to 2020 (Fig. 5). Biochemistry/Molecular Biology, Cell Biology, Neurosciences, and Multi-disciplinary Sciences were the four fields mostly associated with SGs.

### 3.6. Co-cited references

**Figure 6.**
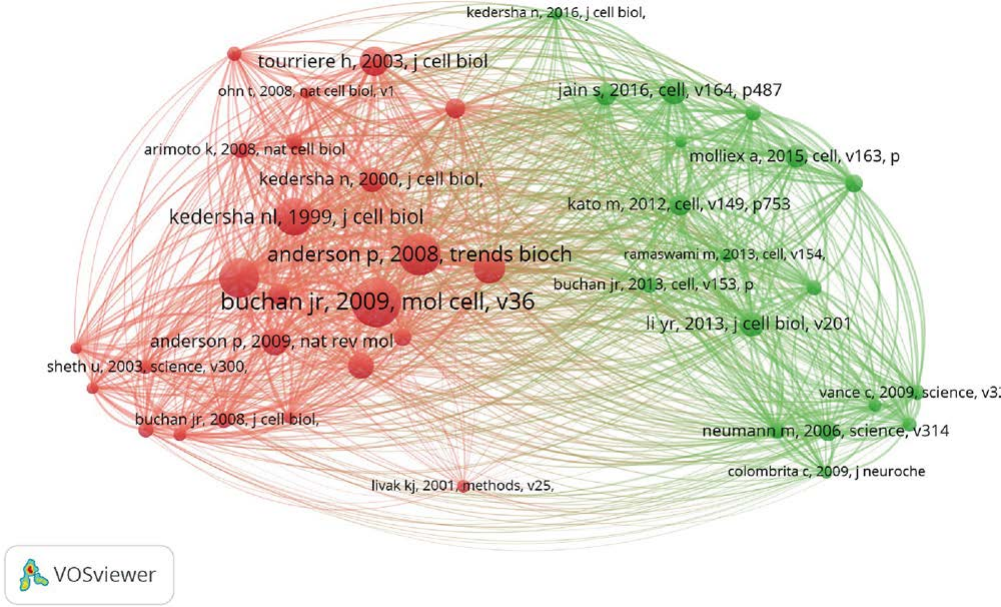
The co-cited reference network of stress granules.

The co-citation relationship means the relationship formed when both articles become references to a third document. The size of the node positively correlated with the reference count (Fig. 6). From 2011 to 2020, the most cited papers related to SGs constituted the background structure of this field. The top 10 references with the highest co-cited counts were summarized (Table 4). The most co-cited reference was “Eukaryotic stress granules: the ins and outs of translation,” an article published by Buchan JR in Mol cell,^[3]^ which mainly focused on SGs and processing bodies (PBs). In addition, it is worthy to note that Kedersha N published 40% of the top 10 references. The 4 articles by Kedersha N focused on the assembly, sorting, and remodeling of SGs. In addition, Kedersha N had a strong cooperative relationship with many scholars with high output in the field of SGs.^[29–32]^

**Table 4:** The top 10 references with the highest co-cited counts.

Citations	Author	Source	DOI	Total link strength
418	Buchan JR	Mol Cell	10.1016/j.molcel.2009.11.020	894
353	Anderson P	Trends Biochem Sci	10.1016/j.tibs.2007.12.003	845
340	Kedersha N	J Cell Biol	10.1083/jcb.200502088	800
316	Kedersha N	J Cell Biol	10.1083/jcb.147.7.1431	883
273	Kilks N	Mol Biol Cell	10.1091/mbc.e04-08-0715	777
250	Tourriere H	J Cell Biol	10.1083/jcb.200212128	697
226	Anderson P	Nat Rev Mol Cell Bio	10.1038/nrm2694	573
223	Jain S	Cell	10.1016/j.cell.2015.12.038	355
223	Kedersha N	Method Enzymol	10.1016/s0076-6879(07)31005-7	528
213	Kedersha N	J Cell Biol	doi 10.1083/jcb.151.6.1257	664

### 3.7. Keyword co-occurrence and burst

**Figure 7.**
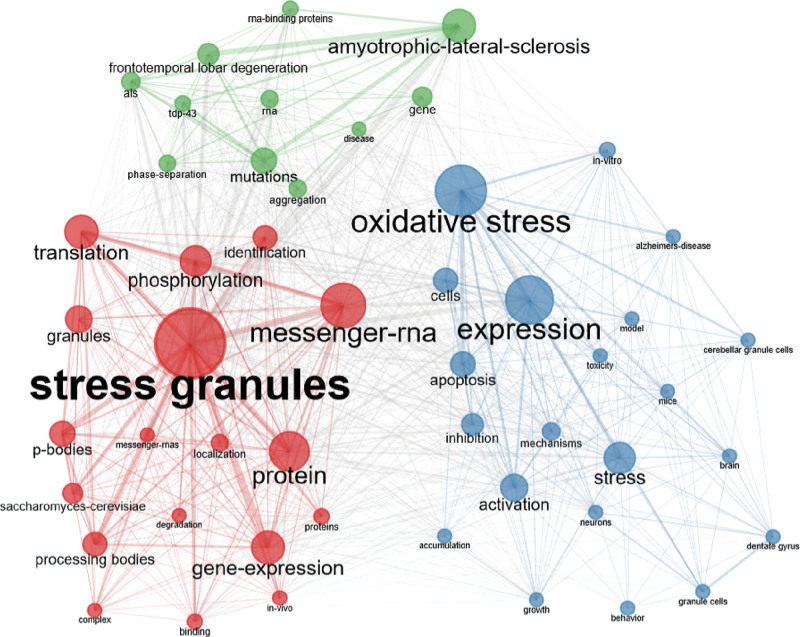
The network of keyword co-occurrence in stress granules research.

Keywords represent the core content of research. Keyword co-occurrence analysis detects the research hotspots in a certain field and burst keywords symbolize research frontiers over a period of time. Bibliometric package for R software was used to establish a knowledge map of keyword co-occurrence (Fig. 7) and to identify the top 20 keywords based on the frequency of SG research from 2011 to 2020. The top keywords were ”stress granules,“ ”messenger-RNA,“ ”protein,“ ”gene-expression,“ and ”translation." The research hotspots on SGs in the past 10 years were generally in the areas of occurrence and outcome of infectious diseases, neurological diseases, atherosclerosis, autoimmunity, cancer development, and other diseases.

**Figure 8.**
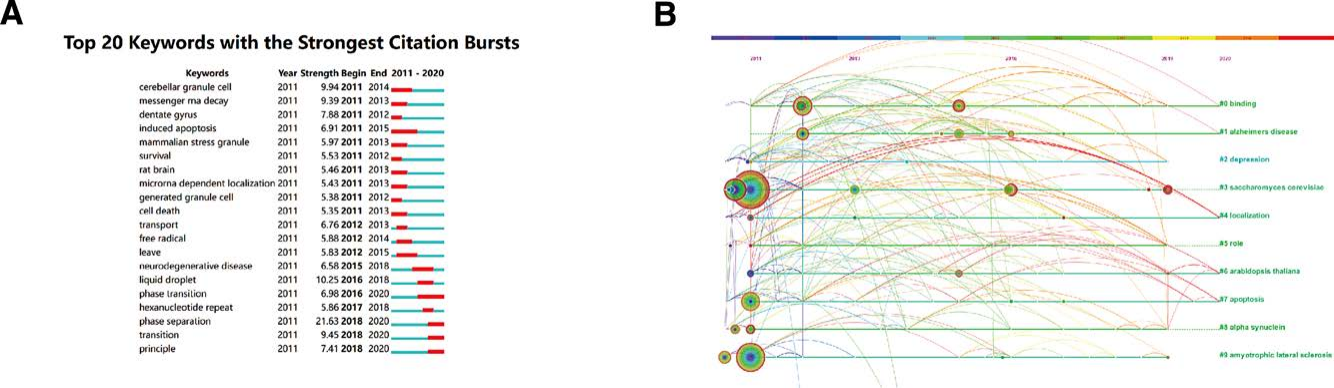
The keywords analysis in the stress granules field. (A)Top 20 keywords with strong citation bursts; (B)The keywords timeline in the stress granules field.

By calculating the frequency of keywords in research on a particular topic, burst keyword detection was conducted to identify research hotspots based on the growth rate of keywords. It can be used to observe emerging theories and themes and frontiers in a certain period of time. In order to better display cutting-edge hotspots, the top 20 articles cited most frequently each year from 2011 to 2020 were selected as the content for analysis. Table 5 lists the keywords with the strongest citation bursts. The keywords with strong bursts before 2015 were “Saccharomyces cerevisiae,” “processing body,” “expression,” “alpha synuclein,” “messenger RNA,” “apoptosis,” “spatial memory,” and “gene expression” (Fig. 8A). The burst keywords after 2015 mainly included “prion like domain,” “liquid droplet,” “phase transition,” “RNA binding protein,” “phase separation,” “domain,” “transition”.

**Table 5:** The top 20 keywords in the stress granules field.

Keyword	Occurrences	Total link	Keyword	Occurrences	Total link
Stress- granules	647	892	Phosphorylation	218	405
Oxidative stress	569	360	amyotrophic-Lateral-sclerosis	214	317
Expression	422	595	Apoptosis	192	299
Messenger-rna	357	593	Cells	187	308
Protein	307	528	Identification	179	281
Stress	297	226	P-bodies	168	327
Gene-expression	272	391	processing bodies	166	321
Translation	229	489	Inhibition	160	269
Activation	227	291	Mutations	151	269
Granules	223	278	Gene	141	227

We drew a timeline diagram of the top 20 most cited articles each year. The lines connecting the different circles indicate the co-citation relationship (Fig. 8B). The 10 cluster labels represent the popular and common topics. The larger the area of the circle, the higher the citation frequency. It can be speculated that the research frontiers of SGs mainly focus on gene expression, RNA regulation, and phase transition.

## 4. Discussion

The research on SGs is currently in full swing; especially, the potential value in clinical medicine has attracted the attention of several researchers. SGs have become a booming research field, and hence, a better understanding of SGs is essential for future research. This study was the first to sort out and summarize the studies on SGs from 2011 to 2020. It mainly used bibliometric methods to analyze the number of publications, co-citation analysis, and keyword analysis.

In the past 10 years, the number of SG-related publications has gradually increased. The development of this field has also attracted the attention of several countries, institutions, and individuals. The United States was also the most influential country in this field of research; the institution with the highest number of publications was Chinese Acad Sci, followed by McGill Univ. The journal with the highest number of publications was PLOS ONE, and its number of publications was approximately twice that of the journal in the second place; among the top 10 journals, the IFs of eight journals were all >5, which shows that the SG field has great potential. The individual author with the largest number of published papers was Parker R, who has published 40 books in 10 years. Another scholar among the top 10 authors who made significant contribution was Anderson P; both Parker R and Anderson P are productive researchers in the field of SGs. It is worth mentioning separately that Parker R is mainly involved in the modification of SGs and the study of the transcriptome of SGs and has published many relevant highly cited articles.

CiteSpace and VOSviewer software were used to construct the SG knowledge map. Keyword co-occurrence and co-citation analyses showed that in the SG research field, DNA metabolism mechanism, gene transcription, and modification pathway regulation were the most studied categories. Burst detection showed that relatively important keywords in the past 10 years included cerebellar granule cell, messenger RNA decay, heat shock, unfolded protein response, liquid droplet, phase transition, phase transition, inflammation, and autophagy. Based on the results of this study, it can be reasonably speculated that the research frontiers of SGs are as follows: mechanism of RNA delay, heat shock, and inflammation. These frontiers illuminate the direction for SG in-depth research and establish the value of in-depth mining.

SG research involves various fields, especially Biochemistry and Molecular Biology. The formation of SGs affects related reactions in vivo in 2 ways. First, because of the high local concentration of some components, the equilibrium of the interacting molecules shifts from the free state to the associated state. For example, during viral infection, SGs absorb many antiviral proteins, including RIG-1, PKR, OAS, and RNase L, and as SGs absorb these proteins, they are gradually phosphorylated, thus enhancing the innate immune response and viral resistance.^[33–35]^ Many viruses proliferate by blocking the assembly of stress particles and promoting the cleavage of proteins in G3BP.^[9]^ Moreover, the formation of SGs may also promote the interaction between mRNAs and translation factors, thus promoting the formation of translation initiation complex.^[36]^ Second, SGs may affect the biological response by limiting the interaction between immobilized components and a large amount of cytoplasm. Previous studies have shown that SGs regulate the cellular signal transduction pathway by isolating the signals of proteins, such as TOR, RACK1, or TRAF2. As SGs can also isolate numerous proteins in the metabolic process of RNA, its formation may have a wider impact on cell physiology. However, the mechanism through which the assembly of SGs fully influences the regulation of mRNA function or other aspects of cell physiology remains to be elucidated.

Through in vitro protein separation simulation experiments, Elbaum et al confirmed that the assembly and depolymerization of SGs are related to the physical properties of related proteins, and this assembly and depolymerization is closely related to the in vivo phase transition.^[37]^ In addition, Emilio et al proved that Tudor staphylococcal nuclease in *Arabidopsis thaliana* is a part of SGs and PBs. Total local citation score is essential for the integrity and function of the cytoplasmic messenger ribonucleic acid complex mRNP. It affects the downstream translation response by affecting the structural integrity of mRNP.^[38]^ As a membrane-free and highly dynamic organelle, SGs are not only a regulator of translation, nuclear balance, and protein balance, but also a signal hub that determine cell viability and pressure recovery. The assembly of SGs depends on the aggregation of intrinsically disordered domains.^[39,40]^ The dephosphorylation of nucleating protein intrinsically disordered domains can promote the liquid phase separation of the system.^[37]^ This physical phase separation phenomenon is related to animal and plant diseases. For example, because SGs exist widely in metastatic and circulating tumor cells,^[36,41]^ external stimulation leads to the aggregation and separation of SGs, which becomes a potential biomarker for diagnosis and prognosis of diseases. Under continuous stress, the ability of some RNA-binding proteins to assemble SGs increased. In stressed transgenic *A. thaliana*, regular SGs were distributed in the cytoplasm of mesophyll cells. Miroshnichenko et al obtained tobacco plants with blocked SG assembly through an antibody-mediated method. Under long-term stress induced by lethal temperature, the cell membrane of tobacco plants blocked by SGs was ruptured, resulting in cell death.^[42]^ Koguchi et al proved by immunoprecipitation and other experiments that the plant vascular zinc finger protein VOZ2 formed SGs under stress, and this process led to the inhibition of intracellular transcription. When the stress was relieved, the transcriptional level was restored, which indicated that SGs were the cytoplasmic particles of mRNA storage and decay under abiotic stress.^[43]^

## 5. Conclusion

To the best of our knowledge, this is the first bibliometric analysis of SGs. This study provides a unique perspective for future research on SGs and predicts the hotspots and directions of future research. This study has its limitations. For example, to ensure the timeliness and accuracy of the study, the time limit was set to nearly 10 years and the article type was set to article, excluding reviews and other article types. In summary, this study used bibliometric methods and software to sort, summarize, and analyze the available literature on “SGs” in the past 10 years. The study found that the United States and China were the dominant countries in this field, and the two countries had strong cooperation. Research hotspots related to SGs included heat shock and mRNA transcription. This study summarizes the frontiers of SGs, including inflammation and transcription modification, and the findings can help future researchers to clarify the research direction and hotspots.

## Author contributions

Conceptualization: Haiyang Yu, Qinhao Chen, Yueyin Pan

Data curation: Haiyang Yu, Qinhao Chen

Formal analysis: Haiyang Yu, Qinhao Chen

Methodology: Haiyang Yu, Yueyin Panf

Software: Haiyang Yu

Visualization: Haiyang Yu, Qinhao Chen

Writing – original draft: Haiyang Yu, Qinhao Chen, Yueyin Pan

Writing – review & editing: Haiyang Yu, Qinhao Chen, Yueyin Pan
